# 20-Fold Increased Limiting Currents in Oxygen Reduction with Cu-tmpa by Replacing Flow-By with Flow-Through Electrodes

**DOI:** 10.1021/acssuschemeng.4c03919

**Published:** 2024-08-12

**Authors:** Nathalie E.G. Ligthart, Phebe H. van Langevelde, Johan T. Padding, Dennis G.H. Hetterscheid, David A. Vermaas

**Affiliations:** † Department of Chemical Engineering, Delft University of Technology, Van der Maasweg 9, 2629HZ Delft, The Netherlands; ‡ Leiden Institute of Chemistry, Leiden University, Einsteinweg 55, 2333 CC Leiden, The Netherlands; § Department of Process and Energy, Delft University of Technology, Leeghwaterstraat 39, 2628 CB Delft, The Netherlands

**Keywords:** hydrogen peroxide, electrolysis, oxygen reduction reaction, mass transfer, flow-through, cell design, flow chemistry

## Abstract

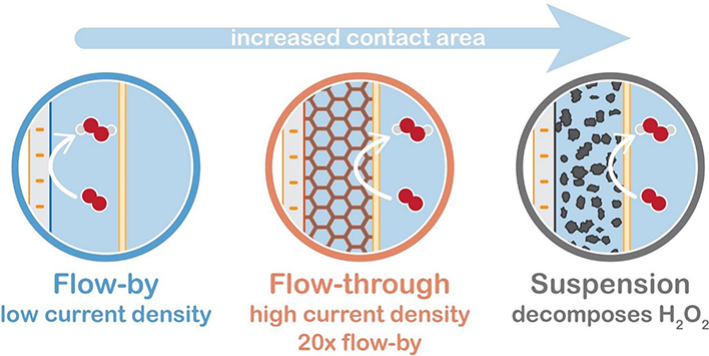

Electrochemical oxygen reduction is a promising and sustainable alternative to the current industrial production method for hydrogen peroxide (H_2_O_2_), which is a green oxidant in many (emerging) applications in the chemical industry, water treatment, and fuel cells. Low solubility of O_2_ in water causes severe mass transfer limitations and loss of H_2_O_2_ selectivity at industrially relevant current densities, complicating the development of practical-scale electrochemical H_2_O_2_ synthesis systems. We tested a flow-by and flow-through configuration and suspension electrodes in an electrochemical flow cell to investigate the influence of electrode configuration and flow conditions on mass transfer and H_2_O_2_ production. We monitored the H_2_O_2_ production using Cu-tmpa (tmpa = tris(2-pyridylmethyl)amine) as a homogeneous copper-based catalyst in a pH-neutral phosphate buffer during 1 h of catalysis and estimated the limiting current density from CV scans. We achieve the highest H_2_O_2_ production and a 15–20 times higher geometrical limiting current density in the flow-through configuration compared to the flow-by configuration due to the increased surface area and foam structure that improved mass transfer. The activated carbon (AC) material in suspension electrodes, which have an even larger surface area, decomposes all produced H_2_O_2_ and proves unsuitable for H_2_O_2_ synthesis. Although the mass transfer limitations seem to be alleviated on the microscale in the flow-through system, the high O_2_ consumption and H_2_O_2_ production cause challenges in maintaining the initially reached current density and Faradaic efficiency (FE). The decreasing ratio between the concentrations of the O_2_ and H_2_O_2_ in the bulk electrolyte will likely pose a challenge when proceeding to larger systems with longer electrodes. Tuning the reactor design and operating conditions will be essential in maximizing the FE and current density.

## Abstract 2

20-fold intensified electrochemical oxygen reduction is demonstrated by utilizing a flow-through cell configuration to allow upscaling of sustainable H_2_O_2_ production.

## Introduction

Hydrogen peroxide (H_2_O_2_) is an important chemical that is widely used in established methods for chemical synthesis,^1^ disinfection,^2^ and bleaching,^3^ as well as applications such as advanced oxidation processes in water treatment,^4^ and fuel cells.^5^ Contrary to the use of H_2_O_2_ as a green oxidant, its anthraquinone production process is energy-intensive and environmentally unfriendly.^6^ The continued and increasing demand^7^ for H_2_O_2_ as a green oxidant has provoked the development of alternative production methods, such as electrochemical 2e^–^ oxygen (O_2_) reduction.

The oxygen reduction reaction (ORR) can run on renewable energy, water, and oxygen as inputs and provides a sustainable route for H_2_O_2_ synthesis,^8^ but suffers from challenges imposed by the low solubility of O_2_ in water (1.1 mM when in contact with pure O_2_ gas at standard conditions). The small amount of O_2_ available near the electrode depletes rapidly when working at higher current densities. This causes severe mass transfer limitations and lowers catalyst selectivity considerably at economically viable current densities.^9,10^ Electrochemical H_2_O_2_ synthesis has been commercialized in the Dow-Huron process^11^ that produces highly alkaline H_2_O_2_ solutions, of which the pH is lowered after production to prevent H_2_O_2_ decomposition and to fit the requirement for acidic or neutral H_2_O_2_ solutions of many applications. Commercially applicable processes for producing neutral and acidic H_2_O_2_ solutions directly are still lacking.^12^ Improved reactor designs are necessary to alleviate mass transfer limitations and advance toward widely applicable practical-scale electrochemical H_2_O_2_ synthesis devices.^5^

Here, we perform ORR in an electrochemical flow cell with three different electrode configurations and Cu-tmpa (tmpa = tris(2-pyridylmethyl)amine) as the catalyst. This catalyst was selected because it is the fastest copper-based molecular ORR catalyst reported to date, catalyzing the reduction of oxygen with more than 2 million turnovers per second.^13^ Furthermore, electrochemical experiments have shown that this Cu-based molecular catalyst converts O_2_ in neutral pH in two consecutive steps, starting with a fast 2-electron reduction of O_2_ to H_2_O_2_, followed by a slower second 2-electron reduction of H_2_O_2_ to H_2_O, therefore generating H_2_O_2_ as a stable intermediate.^13,14^ It has been shown that in a rotating disk electrode (RDE) setup, the ORR selectivity is determined by the local O_2_ and H_2_O_2_ concentrations at the electrode surface, and thus highly dependent on transport of O_2_ toward and H_2_O_2_ away from the electrode.^15^ Cu-tmpa was shown to generate H_2_O_2_ solutions with high Faradaic efficiency (FE) for up to 8 h. However, an RDE setup is a highly controlled and idealized system. Therefore, in this work, we extend Cu-tmpa studies that have been limited to fundamental studies on RDEs to more applicable flow systems with a larger electrode area to obtain insight into the effect of cell configuration on the activity and selectivity of H_2_O_2_-generating molecular catalysts. Additionally, we study the influence of the different electrode configurations and electrolyte flow velocities on mass transfer-limited electroconversion, and we relate our findings to the implications for larger electrodes to provide insights helpful for scaling up electrochemical H_2_O_2_-generating systems.

**Figure 1:**
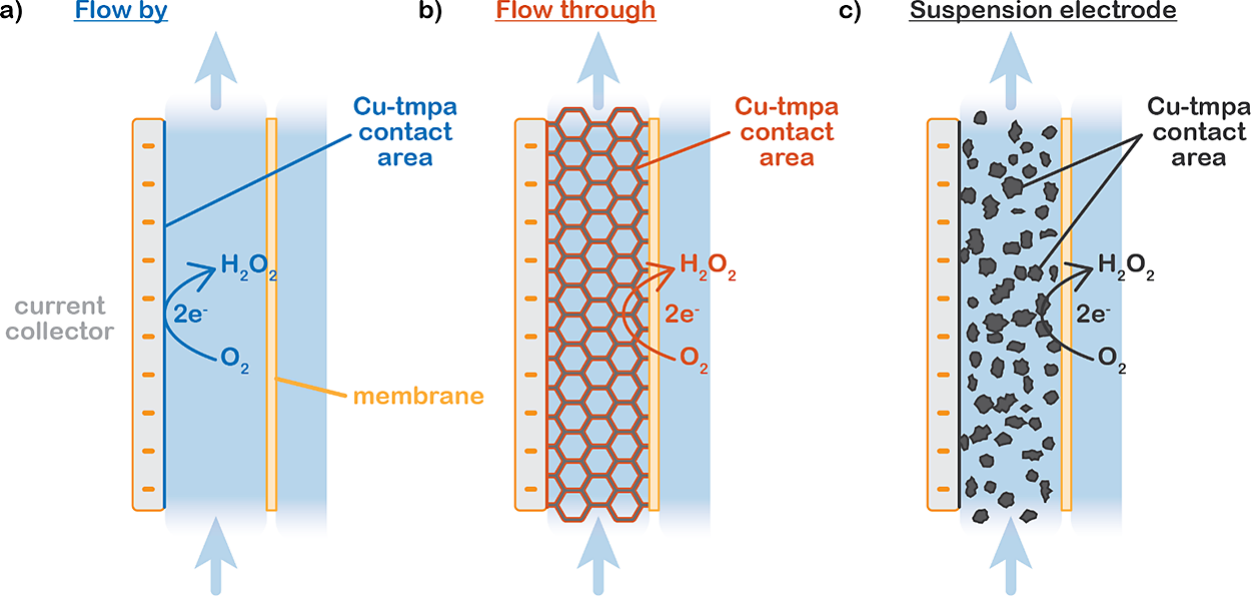
Studied current collector configurations (a) flow-by, (b) flow-through, and (c) suspension electrodes, for homogeneous catalysis of oxygen reduction by Cu-tmpa.

Replacing 2D electrodes by 3D structures can alleviate mass transfer limitations by providing a larger contact area with the electrolyte and shorter mass transport distances.^10,16^ We perform the ORR in a conventional flow-by configuration (2D), as well as in a flow-through configuration and on a flowing suspension electrode (both 3D). The flow-by configuration consists of a flat electrode with the electrolyte flowing along its surface (Figure 1a), and it is likely to develop a thick diffusion boundary layer and severe mass transfer limitations. The flow-through configuration has an electrically conductive foam inserted into the electrolyte flow path (Figure 1b), enlarging the contact area, allowing the electric current to percolate through the entire channel, and hindering the development of a thick boundary layer. The suspension electrode is composed of conductive porous microparticles suspended in an electrolyte and flows along a 2D current collector (Figure 1c). The porous particles provide an even larger surface area than the foam, while conductive networks and particle collisions also allow for electron percolation into the channel.^17−19^

Evaluating the influence of reactor design on mass transfer is essential for achieving the high product selectivity and current densities needed for advancing electrochemical H_2_O_2_ synthesis to an industrially relevant technology.

## Methods

Cu-tmpa was synthesized as described by Langerman et al.^14^ All experiments were performed in the electrochemical flow cell and setup shown in Figure S1. The cathodic and anodic compartments (3 mm thick) were separated by a Nafion 117 cation exchange membrane (CEM) that was soaked and stored in an electrolyte. We used a three-electrode setup, with a glassy carbon plate (Goodfellow) used as cathodic current collector in all experiments, of which an area of 2.4 × 3.4 cm was exposed to the electrolyte. This was combined with a vitreous carbon foam (3.2 mm thickness, 24 pores/cm, 96.5% porosity, surface area 3937 m^2^/m^3^, Goodfellow) cut into the same dimension and inserted into the flow channel to create the flow-through configuration. An Ir-/Ru-oxide coated Ti-sheet (Permascand) was used as the anode. A leak-free Ag/AgCl electrode (LF-1-45, Alvatek) was used as a reference electrode (RE). It was inserted through the side of the flow channel with its tip in front of the glassy carbon plate, as illustrated in Figure S1. A small hole (Ø 3 mm) was made in the carbon foam at this spot to accommodate for the RE insertion.

A phosphate buffer of 0.5 M Na_2_HPO_4_/NaH_2_PO_4_ (≥99.999%, Honeywell Fluka TraceSELECT) at pH 7 was used as catholyte and anolyte to allow for comparison with previous studies,^13−15^ and to provide good Cu-tmpa and H_2_O_2_ stability and sufficient conductivity to achieve acceptable cell potentials. The catholyte was saturated with oxygen by sparging O_2_ gas at 50 mL/min (controlled with mass flow controllers by Bronckhorst) during and for at least 30 min before the experiment. 80 mL of catholyte and anolyte were used in the case of the flow-by and flow-through configurations, 40 g of carbon suspension in 0.5 M phosphate buffer replaced the catholyte, and 40 mL of phosphate buffer was used as anolyte in the suspension electrode experiments. The suspension was prepared by adding 10 or 20 wt % of activated carbon (AC, 20 μm median particle size, 1000 m^2^/g, Norit SX Plus CAT, Sigma-Aldrich) to the phosphate buffer under thorough stirring, followed by 30 min of sonication.

Cyclic voltammetry scans (CVs) were performed at a scan rate of 100 mV/s using an IviumStat (±5 A/±10 V, Ivium) potentiostat (see the Supporting Information for more details). The scans were performed at various flow speeds (peristaltic pump, L/S Precision Pump System, Masterflex) before and between two additions of 5 μM Cu-tmpa to investigate the effect of flow and catalyst concentration on the limiting current.

The ORR performance was evaluated during chronoamperometry (CA) at a cathode potential close to the half-wave potential (E_cat/2_) of 0.3 vs RHE found in previous studies^13,14^ that results in roughly 75% of the peak current. This allowed us to push the current densities toward their maximum without entering the mass transfer-limited regime in the CVs. As the catalytic peaks were not visible in the CV scans of the suspension electrodes, the suspension CAs were run at the potentials selected for the flow-by and flow-through configurations. Each CA was run for 1 h at a flow velocity inside the catholyte channel of 19 mm/s when using the flow-by and flow-through configurations, and at a lower flow velocity of 9 mm/s when using a suspension electrode to prevent operational complications such as clogging over the course of the experiment. The H_2_O_2_ concentration was measured periodically with a reflectometer (RQflex 20, Merck) and corresponding peroxide test strips (0.2–20.0 mg/L H_2_O_2_). The accuracy of the test strips for measuring peroxide has been verified in ref.^15^ The samples were diluted with a buffer to fit the detection window of the test strips whenever necessary. The precise reaction conditions of each experiment are listed in Table S1.

The stability of H_2_O_2_ in the different experimental conditions was tested by adding a known amount of H_2_O_2_ and measuring the concentration in the liquid at time intervals. In the case of the flow-by and flow-through configurations, the flow cell was assembled as described above. The anolyte compartment was filled with electrolyte and closed off, while 72 g of buffer was cycled through the catholyte compartment at 40 mL/min (9 mm/s inside the channel). In the case of the suspension electrode, the H_2_O_2_ concentration was monitored after addition to 80 g of the 10 wt % AC suspension under continuous stirring inside a glass bottle. The first sample was taken 30 s after each addition and passed through a filter (Whatman Puradisc H-PTFE syringe filters, 0.2 μm, hydrophilic) to remove the AC before measuring the H_2_O_2_ concentration with the reflectometer. Taking the sample and filtering took about 10 min.

A similar experiment was performed to investigate whether H_2_O_2_ was decomposed or adsorbed by the AC particles. Known amounts of H_2_O_2_ were injected into the 10 wt % AC suspension inside a gastight bottle, and gas samples were taken and analyzed with a gas chromatograph (GC, CompactGC4.0, Interscience) to track O_2_ evolution.

## Results and Discussion

### Flow-Through Configuration Improves the H_2_O_2_ Production

**Figure 2:**
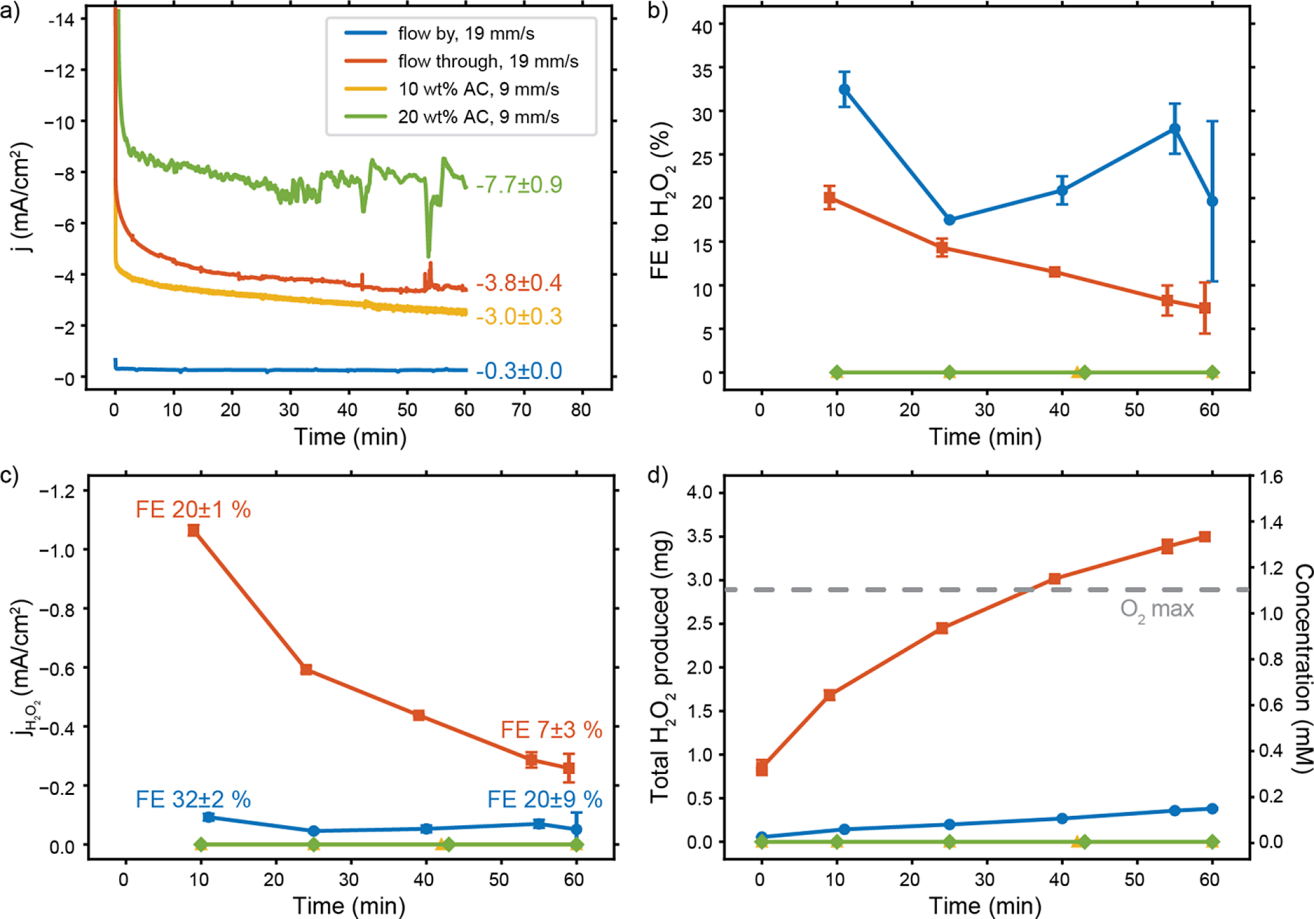
Overall performance of O_2_ reduction by 10 μM Cu-tmpa in 0.5 M phosphate buffer (pH 7) on flow-through, flow-by, and suspension (10 and 20 wt % AC) electrodes. (a) Chronoamperometry showing the differences in achieved current densities through time, performed at 0.31 V vs RHE for the flow-by and 10 wt % suspension, and 0.21 V vs RHE for the flow-through and 20 wt % suspension. The 20 wt % AC graph has been smoothed through a running average over 30 s to remove excessive noise. (b) Measured FE toward H_2_O_2_ production, and (c) achieved partial current density to H_2_O_2_ (*j*_H_2_O_2__) through time. (d) Resulting cumulative H_2_O_2_ production over time in mg and mM. The H_2_O_2_ concentration exceeds the maximum O_2_ concentration after 36 min of operation in the flow-through configuration.

We measured the current density of the ORR and concentration of produced H_2_O_2_ in the electrolyte during 1 h of CA for comparison of the electrochemical performance in each configuration. Figure 2a shows a significant difference in the achieved current densities among the different configurations, with the 20 wt % AC suspension reaching the highest current density of −7.7 ± 0.9 mA/cm^2^, about twice the current density reached in the 10 wt % AC suspension (−3.0 ± 0.3 mA/cm^2^) and the flow-through electrode (−3.8 ± 0.4 mA/cm^2^), and almost 26 times higher than the flow-by configuration, which reaches only −0.3 ± 0.0 mA/cm^2^.

In addition, we observe a steady decrease in the current density in all systems during CA, especially those operating at higher current densities. The FE decreases over time as well, in the flow-by and flow-through configurations (Figure 2b). Both effects decrease the H_2_O_2_ formation rate over time. We suspect that this is due to two complications. First, the decrease in selectivity over time can be caused by the increasing concentration of H_2_O_2_, both in the reservoir and through the height of the cell, leading to increased Faradaic over-reduction of the produced H_2_O_2_ to form H_2_O and resulting in a lower measured FE toward H_2_O_2_. This is especially an issue in the flow-through configuration, in which the H_2_O_2_ concentration increases most severely and even exceeds the O_2_ concentration after about 36 min of operation, as shown in Figure 2d. While such concentrations generally do not lead to a loss in FE when using an RDE,^15^ H_2_O_2_ accumulation has been shown to decrease selectivity in flow cells.^20^ The produced H_2_O_2_ spends a significantly longer time near the electrode before getting diluted inside the reservoir, compared to a setup using an RDE. This essentially increases the risk of H_2_O_2_ reduction. Second, the overall decrease in current density over time can be caused by slow dissolution of O_2_ in the reservoir. To illustrate, all dissolved O_2_ will be consumed within 9 min at the ORR rate reached in the flow-through configuration if no fresh O_2_ is supplied (see Supporting Information for calculation). Reaching a sufficiently high dissolution rate to keep up with the O_2_ consumption and maintain the maximum O_2_ concentration is challenging and unlikely when using a simple sparger for saturation.^10^ Both the O_2_ depletion and H_2_O_2_ accumulation contribute to altering the O_2_/H_2_O_2_ ratio near the electrode and affect the ORR and the hydrogen peroxide reduction reaction (HPRR) rates according to the ORR rate = *k*_ORR_[Cu-tmpa][O_2_] and HPRR rate = *k*_HPRR_[Cu-tmpa][H_2_O_2_], increasing the HPRR rate during the CA. In turn, this also lowers the overall current density because *k*_HPRR_ is an order of magnitude lower than *k*_ORR_.^13^ The combination of these two effects ultimately leads to a more severe loss in partial H_2_O_2_ current density in the flow-through than in the flow-by case (Figure 2c).

Despite the lower selectivity, the flow-through configuration displays the highest partial H_2_O_2_ current density at all times (Figure 2c). The higher H_2_O_2_ production rate results in almost 10 times more H_2_O_2_ being produced in the flow-through configuration than in the flow-by configuration within 1 h of operation (Figure 2d). The flow-through setup produces concentrations in the mM range, which is already sufficiently high for applications like H_2_O_2_/UV disinfection.^21^

Although the total current densities achieved in the suspension electrodes almost match (for 10 wt % AC) or even surpass (for 20 wt % AC) the total current density reached in the flow-through configuration, no H_2_O_2_ was detected in the suspensions. We suspect that the suspensions are interfering negatively with the reaction because the glassy carbon plate used in the flow-by system is also present here and was expected to allow for at least some H_2_O_2_ production. We will address this issue later.

### Electrode Configuration and Flow Conditions Enhance Mass Transfer

**Figure 3:**
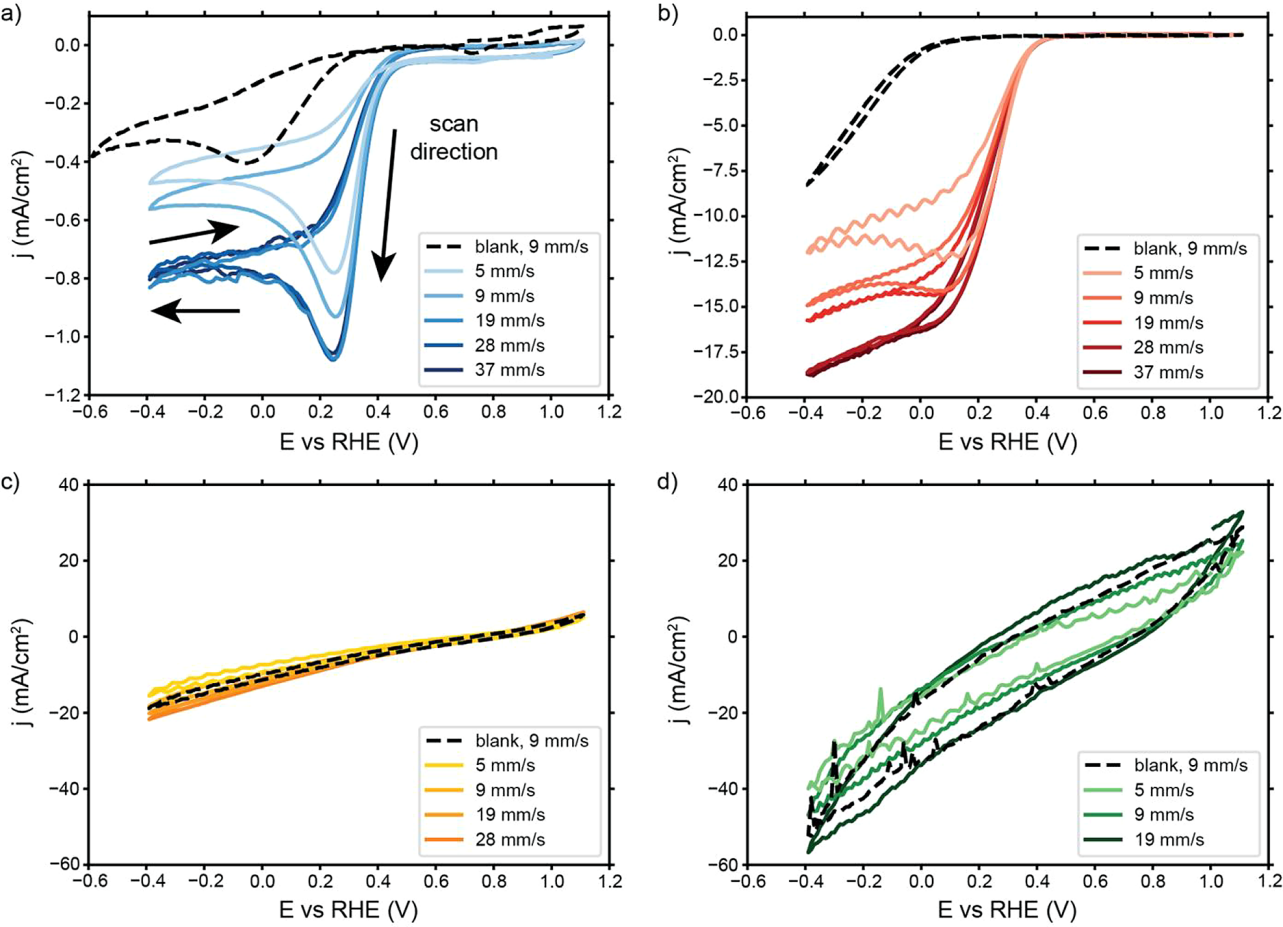
CV scans of 5 μM Cu-tmpa in O_2_-saturated 0.5 M phosphate buffer of pH 7 under various flow velocities with (a) glassy carbon plate (flow-by), (b) glassy carbon foam (flow-through), (c) 10 wt % AC suspension, and (d) 20 wt % AC suspension electrodes, at a scan rate of 100 mV/s. The black dashed lines correspond to blank measurements without any Cu-tmpa present. The blank measurement in the flow-by shows an extra reduction peak at an earlier onset potential or ORR on glassy carbon than in the flow-through configuration due to a contaminant. Nevertheless, addition of Cu-tmpa clearly increases the current and decreases the onset potential in the flow-by and flow-through systems but not in the suspensions. Current fluctuations are visible in some scans; we suspect these are caused by the pulsed flow from the peristaltic pump.

We performed CV scans to further study what current densities can be reached in our H_2_O_2_ synthesis systems and how this relates to the applied potential and the electrolyte flow rate. The CV scans for the different electrodes and flow velocities are shown in Figure 3. The current increases most sharply between roughly 0.4 and 0.2 V vs RHE, depending on the configuration. This is in line with previous studies, wherein the half-wave potential (E_cat/2_) was found at 0.3 V vs RHE.^13,14^

First we consider the CVs of the flow-by (Figure 3a) and flow-through (Figure 3b) cases. The flow-by configuration leads to a typical peak-shaped CV with a peak current in the forward scan caused by the transition from saturated O_2_ conditions at the electrode surface to the formation of a depleted diffusion boundary layer. The peak is followed by a plateau toward the cathodic vertex, where the O_2_ at the surface has already been depleted and the current is limited by O_2_ transport from the freshly delivered bulk electrolyte. Increasing the flow velocity increases the peak current and plateau current to a certain extent by lowering the residence time and reducing the boundary layer thickness, respectively.

The flow-through configuration (Figure 3b) reaches a significantly higher geometrical current density of 5–15 times, depending on the flow rate, compared to the highest peak current in the flow-by configuration (Figure 3a). The active surface area per flow cell area is increased 13.5 times by the porosity of the flow-through electrode compared to the flow-by plate electrode (see Supporting Information for calculation). This suggests that most of the increase in the geometric current density is due to the larger active surface area. However, the difference in CV shape reveals that the mass transfer conditions also depend on the electrode systems. The lowest flow velocity (5 mm/s) for flow-through results in a similar shape as we have seen in the flow-by configuration but with a less pronounced peak at 0.25 V. The peak shape transforms to resemble an S-shape voltammogram with increasing flow velocity and is completely invisible when applying higher flow velocities (28 and 37 mm/s). The plateau shape in the CV indicates that a constant O_2_ supply is available, which corresponds with O_2_ transport from the bulk instead of O_2_ from a transient boundary layer build-up. The transition toward steady-state diffusion (S-shape) at relatively low flow velocities suggests that the boundary layer in the flow-through is much thinner than in the flow-by case. This makes sense, as the 3D character of the foam forces a frequent restart of the boundary layer development along the length of the channel. Therefore, the foam reduces the boundary layer thickness compared to the flow-by system, in which the diffusion boundary layer continues to develop along the entire channel length, resulting in an ever-increasing boundary layer thickness along the current collector surface. We estimate the average boundary layer thickness (δ) in the flow-by configuration at 180 μm, versus 11 μm in the flow-through configuration, using , with *D* the diffusion coefficient of O_2_ in water, and *k* the mass transfer coefficient.^22,23^ We obtain the mass transfer coefficients of a plate and foam, *k*_plate_ and *k*_foam_, respectively, later with eqs 3–5.

Although suspension electrodes give even higher current densities compared with the flow-by and flow-through configurations (Figure 3c,d), the ORR performance cannot be compared from these scans. The steep slope of the ORR that is expected below 0.6 V vs RHE is clearly present in the other flow configurations but is not visible when using the suspension electrodes, and we do not see a difference between the experiments in the presence or absence of catalyst. The high currents seem to be due to the high capacitance of the suspensions, caused by the large surface area of the porous carbon particles. This agrees with the increase in hysteresis when comparing the 10 wt % (Figure 3c) with the 20 wt % (Figure 3d) AC suspension, as the increase in carbon loading raises the capacitance for two reasons: (1) a higher carbon loading results in a larger surface area, and (2) a higher carbon loading also gives higher conductivity and more percolation into the bulk of the electrode, making more surface area accessible for capacitive charging.^24^ The large capacitance of the suspensions makes it impossible to observe an onset potential for the ORR in CVs and shows that at least part of the current during CA can be attributed to electric double layer (EDL) charging.

### Catalyst Concentration and Stability

**Figure 4:**
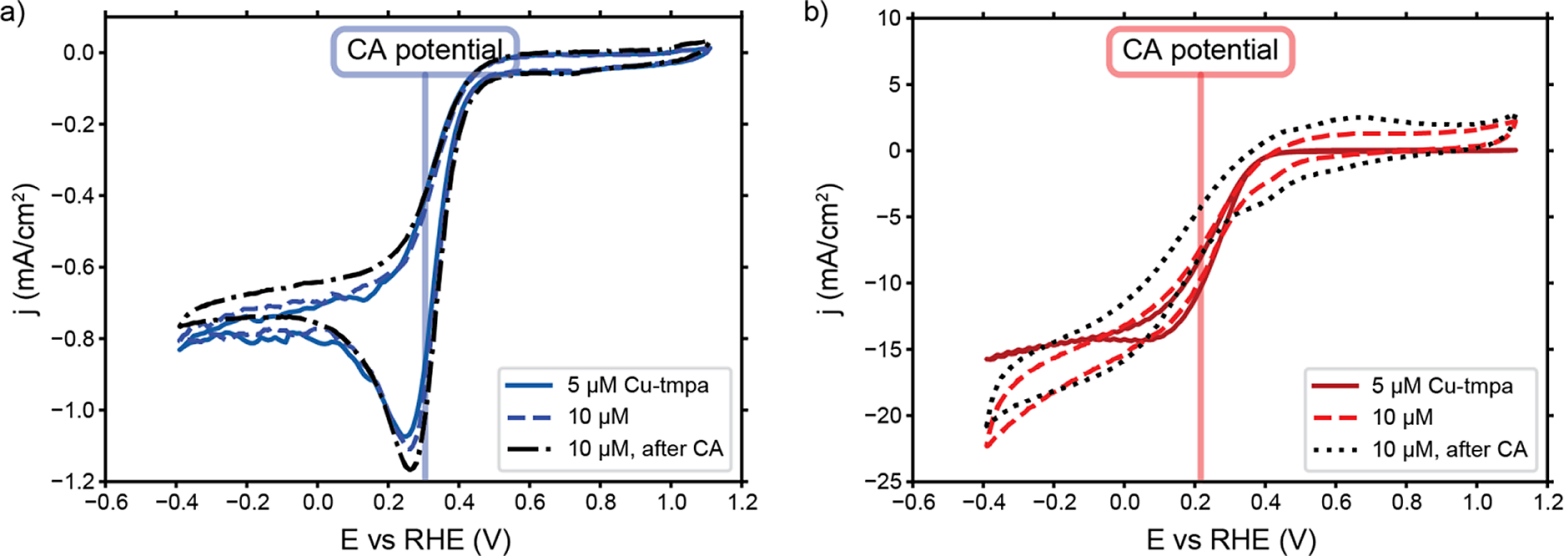
Influence of Cu-tmpa concentration and 1 h of chronoamperometry (CA) on CV scans at a scan rate of 100 mV/s and an electrolyte flow velocity of 19 mm/s with a (a) glassy carbon plate (flow-by) and (b) glassy carbon foam (flow-through) as current collectors. The vertical line indicates the potential applied during CA.

We performed CVs with Cu-tmpa concentrations of 5 and 10 μM to check whether the current is limited by catalyst availability in addition to O_2_ availability. Doubling the Cu-tmpa concentration had no significant effect on the reached currents in any of the configurations, as shown in Figure 4a,b for the flow-by and flow-through configurations, respectively (see Supporting Information for the suspensions). This is in line with observations inside this concentration range in RDE systems.^15^ The altered shape of the scans performed on the flow-through with 5 versus 10 μM Cu-tmpa was caused by a short contact loss of the RE while scanning the positive potentials, etching the foam surface, and causing a larger EDL to become visible in the CVs. The potential range of interest remained unaffected, and we conclude that neither system is limited by the low Cu-tmpa concentration.

In addition, we performed CVs after 1 h of catalysis (“after CA” curves in Figure 4) at the indicated CA potentials to check the catalyst stability. The shape and reached currents are very similar to those before the CA, suggesting that loss of catalytic activity or Cu-tmpa degradation is not likely cause for the FE loss through time observed in Figure 2. Cu-tmpa reached a turnover number (TON) of 130 during the experiments, which is likely considerably lower than the maximum TON Cu-tmpa can reach in catalysis of the ORR. Previous studies have shown that a TON of at least 250 is achievable.^15^ However, very subtle differences can be observed in the CVs. The slight increase in slope in the flow-by configuration is caused by an increase in the available reactant in the form of H_2_O_2_, and the slight broadening of the flow-through CV can indicate further etching of the foam surface during additional CVs before catalysis. The suspension CVs (Figure S2) show a decrease in current after catalysis, which is in line with the capacitive nature of the suspensions and our observations from Figure 3c,d.

### AC Suspensions Break Down H_2_O_2_

**Figure 5:**
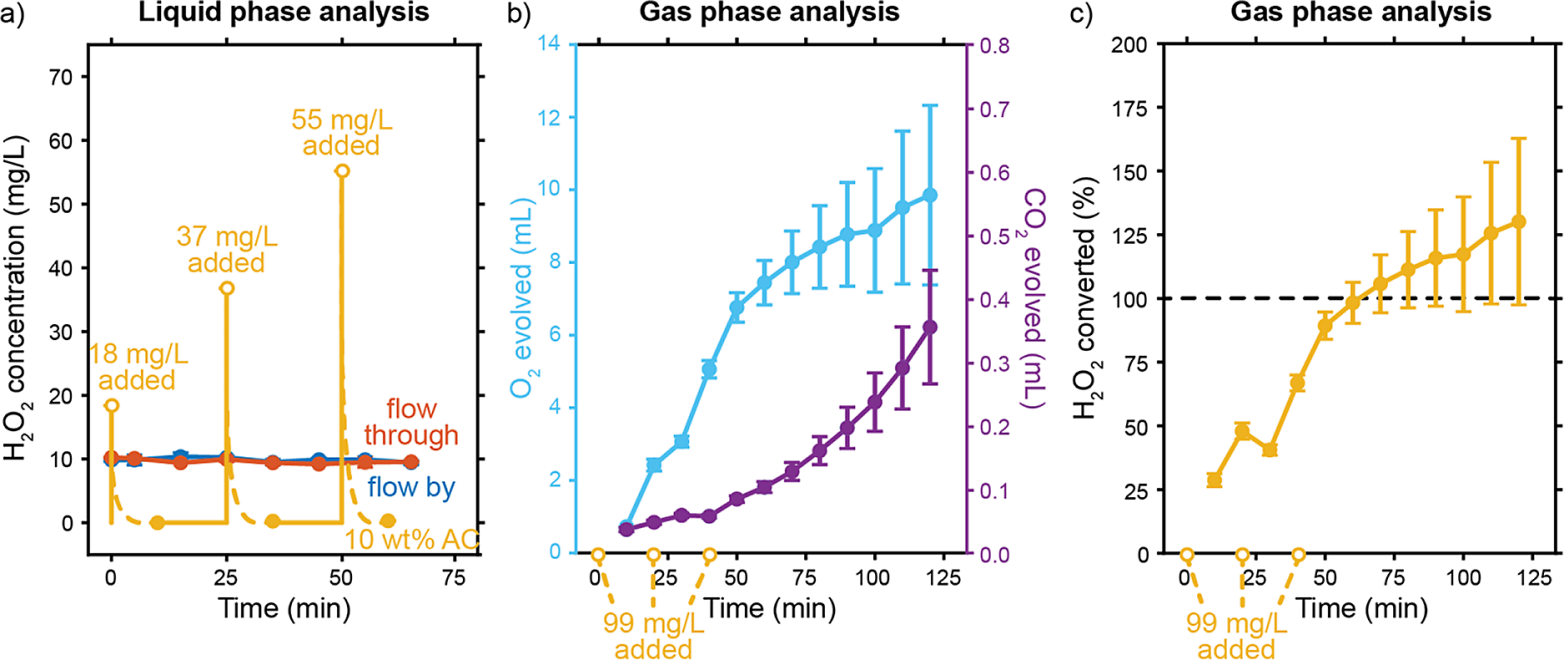
Results showing the 10 wt % activated carbon (AC) suspension breaking down H_2_O_2_ in solution. (a) Measured H_2_O_2_ concentrations through time after one addition of 10 mg/L H_2_O_2_ in case of the flow-by, flow-through case, and after additions of 18, 37, and 55 mg/L H_2_O_2_ to the 10 wt % AC suspension case, spaced 25 min apart. H_2_O_2_ disappears before the concentration in the liquid phase of the suspension can be measured. (b) O_2_ evolution through time after adding H_2_O_2_ to the 10 wt % AC suspension. Smaller amounts of CO_2_ evolve, as well. (c) Percentage of the H_2_O_2_ added that has been decomposed by the 10 wt % AC suspension, as calculated from the evolution of the O_2_ through time.

Even though the AC suspensions show a non-Faradaic response due to their large capacitance, the applied potential was equal to the flow-by and flow-through cases and sufficient for catalysis of the ORR. However, we did not detect any H_2_O_2_ production when applying a potential for a long period of time (Figure 2). To investigate this further, we studied the stability of H_2_O_2_ in a stirred suspension of 10 wt % AC without a potential applied. We added known concentrations of H_2_O_2_ to the suspension and measured the concentration in the electrolyte within 10 min after each addition (Figure 5a). No H_2_O_2_ was detectable in any of these measurements, showing that it vanishes rapidly after addition to an AC suspension via a non-Faradaic process. In contrast, no H_2_O_2_ loss was observed while pumping an electrolyte with known H_2_O_2_ concentrations through the flow-by and flow-through configurations.

We performed the same experiment in a gastight bottle and analyzed the evolving gases to determine whether the suspension decomposes or adsorbs the added H_2_O_2_ on its large surface area. Gas-phase analysis showed the formation of significant amounts of O_2_ upon addition of H_2_O_2_ to the suspensions (Figure 5b). This shows that the H_2_O_2_ disproportionates on the AC particles via 2 H_2_O_2_ → 2 H_2_O + O_2_. Figure 5c shows how much of the added H_2_O_2_ has decomposed over time and that the AC particles can break down all added H_2_O_2_ within 2 h. Note that the concentration added in this experiment is 6 times higher than the eventual concentration formed in 1 h of catalysis in the flow-through configuration, according to Figure 2. In addition, we also detected smaller amounts of CO_2_ forming, which can indicate the oxidation of the carbon particles by H_2_O_2_.

Hence, we have shown that the AC suspensions would decompose any H_2_O_2_ produced by the ORR through non-Faradaic disproportionation, in addition to the already competing Faradaic HPRR. The fast H_2_O_2_ decay prevents us from monitoring how much is produced in the suspension electrodes during CA (Figure 2a). Although the suspension electrodes rival (10 wt % AC) and even surpass (20 wt % AC) the flow-through configuration in terms of achieved total current density, we conclude that either no H_2_O_2_ was produced on the suspension electrodes, or any produced H_2_O_2_ was rapidly decomposed on the suspension particles’ large surface area. Therefore, the AC particles used in this study are not suitable for use in H_2_O_2_ production systems. As a result, the envisaged advantages of using the suspension electrode configuration depicted in Figure 1c could not be realized in this work.

Nevertheless, carbon-based ORR catalysts have been studied extensively in literature and are considered to have great prospects.^8,25^ Suspensions of carefully selected carbon materials may not exhibit the issues we encounter here. Materials such as carbon black (CB), carbon nanotubes (CNTs), and graphene-based materials are commonly used in suspension electrodes,^17,26,27^ and can be modified to act as ORR catalyst^8,9^ as well. Such modified CB, CNT, and graphene-based materials would be promising to test as suspension electrodes for H_2_O_2_ production. Alternatively, a small amount of flowing carbon-based catalyst can be used in combination with a conductive foam to replicate the flow-through configuration with a heterogeneous catalyst instead of Cu-tmpa.

### Comparing Limiting Current Densities to Sherwood Correlations

Having established that a flow-through electrode offers a major advantage in mass transport compared to flow-by electrodes, we can further study the physical cause of the higher limiting current densities. The electrolyte flow velocity and channel properties influence the limiting current density (*j*_lim_) of a mass transfer limited reaction through the Sherwood number (Sh). A higher Sherwood number represents an increased mass transfer coefficient (*k*) and a decreased diffusion boundary layer thickness (δ), leading to a higher limiting current density through eqs 1–3,^22,23^
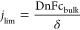
1
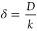
2
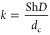
3in which *D* is the diffusion coefficient, *n* the number of electrons involved in the reaction, *F* the Faraday constant, *c*_bulk_ the reactant concentration outside the diffusion boundary layer, and *d*_c_ the characteristic length.

**Figure 6:**
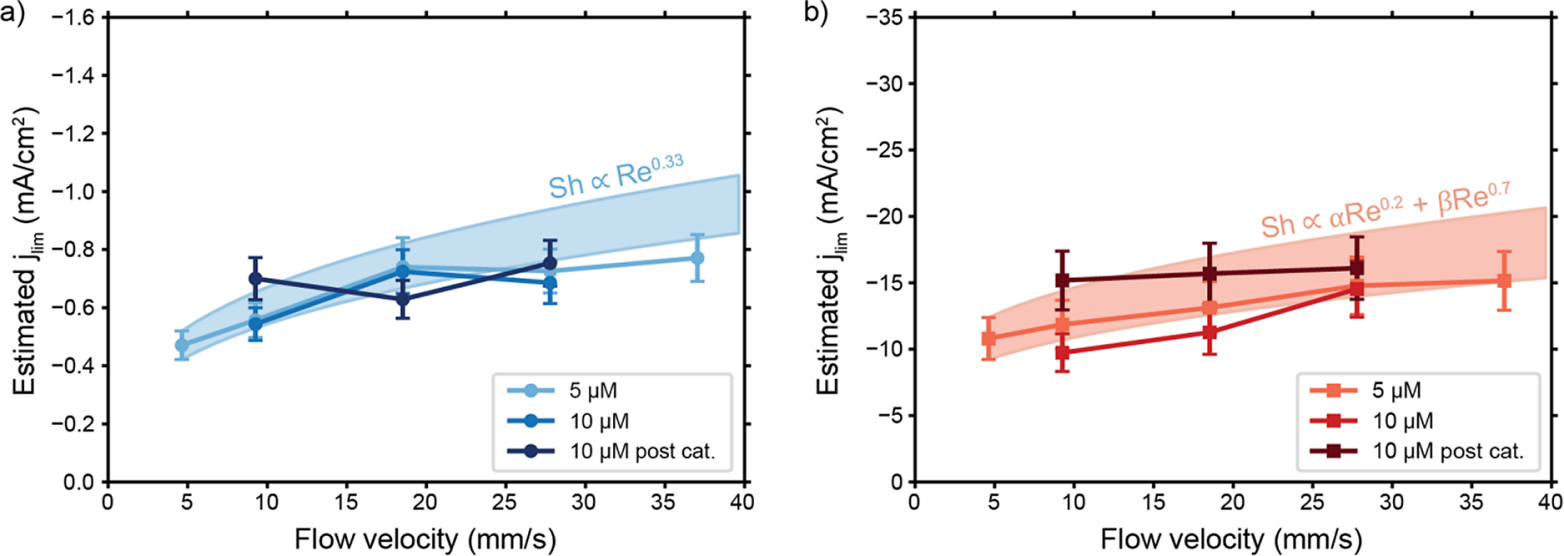
Estimated current densities in the (a) flow-by and (b) flow-through configurations at various flow velocities and catalyst concentrations obtained from CV measurements recorded before and after 1 h of chronoamperometry. Our method for estimating the limiting current densities and the errors is included in the Supporting Information. The shaded areas indicate the expected increase in *j*_lim_ of the 5 μM line with flow velocity based on Sh ∝ Re^0.33^ and Sh ∝ αRe^0.2^ + βRe^0.7^ as indicated.

The Sherwood number along a planar electrode in laminar flow with a fully developed hydrodynamic boundary layer is given by^23^
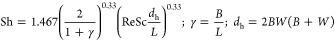
4in which γ is the aspect ratio of the electrode, and *d*_h_ is the hydrodynamic diameter of the channel. These are determined by the electrode length (*L*), electrode and channel breadth (*B*), and channel depth (*W*). The Sherwood number of flow-through of a porous electrode can be estimated with^28^
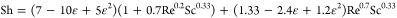
5where ε is the porosity. Both expressions make use of the Reynolds (Re) and Schmidt (Sc) numbers, given by eqs 6 and 7, respectively.
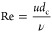
6
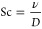
7which include the following equations: (a) the superficial flow velocity (*u*), the kinematic viscosity (ν) of the electrolyte, and a characteristic length scale *d*_c_. The value of *d*_c_ is defined as *d*_h_ (hydrodynamic diameter) for the flow-by configuration and *d*_*s*_ (typical strut size) for the flow-through electrode. We use eqs 4 and 5 to obtain the Sherwood numbers at relevant flow velocities in the flow-by and flow-through configurations, respectively. The only flow rate-dependent term in both equations is the Reynolds number, so we expect Sh ∝ Re^0.33^ in the flow-by electrode and approximately Sh ∝ αRe^0.2^ + βRe^0.7^ in the flow-through electrode. Figure 6 shows the limiting current density at various flow velocities as estimated from CV scans (see Supporting Information for the method). We compare the experimental results with the limiting current densities expected from the obtained Sherwood numbers via
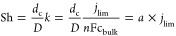
8by fitting them to the limiting current density in the 5 μM Cu-tmpa solution at the lowest flow velocity (5 mm/s) via the factor *a*. The shaded areas in Figure 6 indicate the expected limiting current densities. Both configurations follow the predicted trends with Sh ∝ Re^0.33^ and Sh ∝ αRe^0.2^ + βRe^0.7^ quite well. For the flow-through case, the dominance of the first term (α ≈ 15.3β) gives a weaker flow rate-dependence of approximately Sh ∝ αRe^0.2^ in the low Re laminar flow regime compared to the flow-by electrode. The flow-by electrode benefits more from increased flow because of the severe diffusion boundary layer development over the electrode length, while the flow-through electrode keeps a thinner boundary layer already at low flow velocities because of the shorter developing lengths along the individual struts.

The second term of eq 5, for the flow-through electrode, will get larger than the first term when the Reynolds number exceeds 234, which mitigates the flattening of the curve. This situation is unlikely to occur in channels with dimensions that are typically used in electrolyzers. To illustrate, flow velocities of 5–40 mm/s give Reynolds numbers of only 1–8 in our flow cell. The threshold for Re can be lowered by using a flow-through electrode with lower porosity, but that will add to the pressure drop and pumping costs, as will increasing the flow rate.

Overall, we have shown that the type of current collector configuration (flow-by versus flow-through) can be used to boost the limiting current 15–25 times (see Figure S5). The current density can be raised further by increasing the flow velocity, and the achieved improvement fits well with our expectations from Sherwood correlations. However, flowing faster boosts the limiting current to a much smaller extent than does the electrode shape. Therefore, changing the electrode design is more effective than changing the flow rate.

### Implications for Scale Up

Although the flow-through configuration alleviates mass transfer limitations on the microscale, the low amount of O_2_ available in the bulk of the electrolyte can pose an issue through depletion of the O_2_ along the height of the cell, especially when extrapolating to larger electrolyzers. The local current density is dependent on the local O_2_ concentration and will thus decrease along the height of the channel as O_2_ is consumed and the bulk concentration decreases. We derive the O_2_ concentration profile from the microscopic mass balance (derivation available in the Supporting Information, Section 4.2), which results in
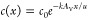
9in which *c*_0_ is the inlet concentration, and *A*_V_is the specific surface area of the foam. The local geometric current density is given by
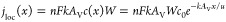
10and can be integrated along the electrode length to yield the average geometric current density (*j*_avg_)
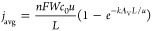
11

We use eq 11 to extract an experimental mass transfer coefficient (*k*_u = 0.019_^exp^) at a flow velocity of 19 mm/s by inserting the average achieved current density over the first 5 min of CA. We estimate the mass transfer coefficient for various flow velocities by multiplying the theoretical mass transfer coefficient (*k*^th^), obtained from eqs 3 and 5, with a factor *k*_u = 0.019_^exp^/*k*_u = 0.019_^th^ to correct for the discrepancy between theory and our experimental setup.

**Figure 7:**
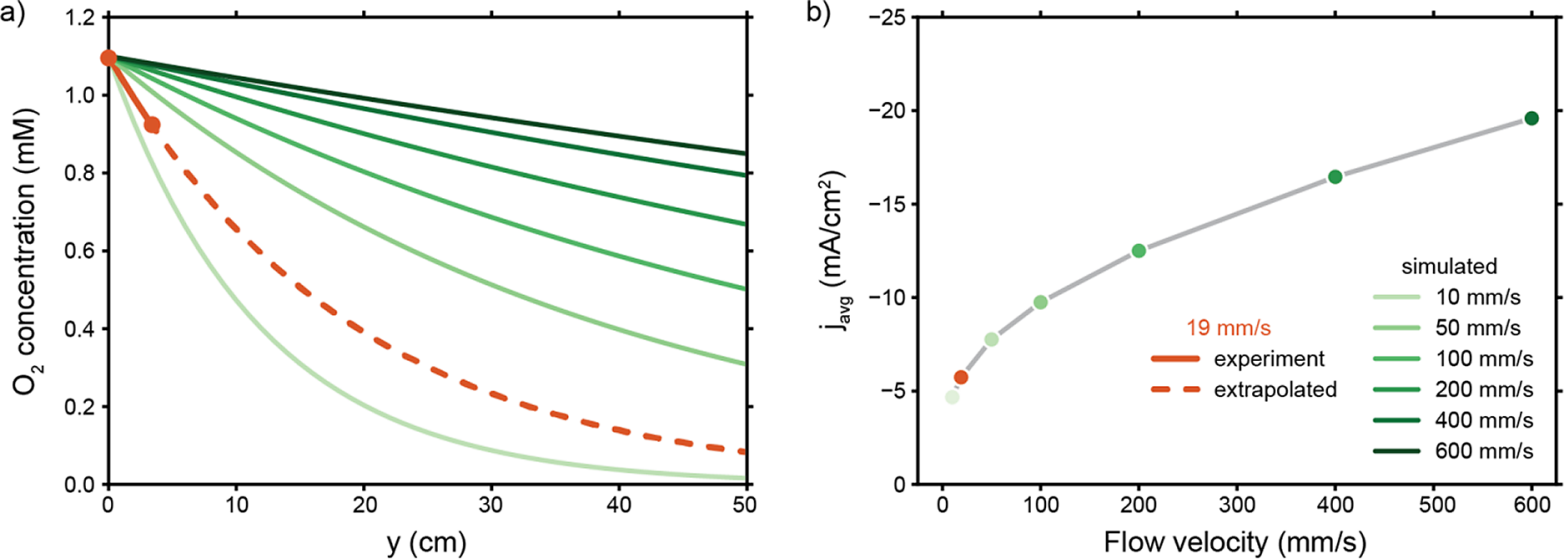
(a) Estimated O_2_ concentration along the height of the flow cell at various flow velocities (see legend in b). (b) Average current density over a 50 cm long electrode at various flow velocities. Both estimates are based on Sherwood numbers and the average geometric current density of 5.7 mA/cm^2^ during the first 5 min of catalysis on a 3.4 cm long electrode.

We evaluate a system with an FE for the ORR of 100%, and we study the influence of flow velocity on the local O_2_ concentration and the resulting average geometric current density. We calculate the concentration profile with eq 9 (Figure 7a) along the 3.4 cm long flow-through electrode (solid orange line) used in our experiments and use this to extrapolate the O_2_ concentration expected for much longer electrodes (dashed orange line). The O_2_ concentration decreases significantly along a 50 cm long electrode and results in an outlet concentration of only 0.1 mM, versus an inlet concentration of 1.1 mM. The ORR current density is highly dependent on the local O_2_ concentration and will thus decrease accordingly higher up in the channel. In turn, O_2_ depletion along the electrolyzer channel lowers the average geometric current density (Figure 7b). Therefore, even a system with perfect selectivity will be limited by the amount of O_2_ as it is scaled up.

Increasing the flow velocity improves the situation considerably in terms of preventing O_2_ depletion along the channel (Figure 7a) and results in a higher average geometric current density (Figure 7b). The higher flow velocity increases the limiting current density in two ways; first, the higher flow rate supplies more fresh electrolyte, which keeps the O_2_ concentration high. Second, the higher flow velocity increases the mass transfer coefficient. Despite this double effect, the gain in local O_2_ concentration and average current density is the largest at relatively low flow velocities. This follows from the exponential term in eq 11, representing the limit of fresh electrolyte (i.e., the limited inflow concentration). As an example, increasing the flow velocity from 19 to 100 mm/s raises the minimum O_2_ concentration 5 times and doubles the average current density, while increasing the flow velocity to 600 mm/s (a factor 30 higher than 19 mm/s) increases the current density only 4 times and induces a significant pressure drop.

The balance between the increased performance and increased pumping costs with the increased flow velocity should be optimized in any electrochemical flow system. As a result, typical flow velocities in comparable applications with single phase flow, such as electrodialysis, are in the order of 10–150 mm/s.^29^ The *j*_avg_–curve in Figure 7b is also steepest in this region, and we expect similar flow velocities to be relevant in our flow-through system, with the most favorable trade-off between increased geometric current density and pumping costs in this regime. We estimated that the theoretical pumping power is already 34% of the electrochemical power at a flow velocity of 600 mm/s in a single-pass (calculation in Supporting Information), which makes it unpractical to flow at such high velocities.

In addition, the electrolyte flow velocity and current density control the H_2_O_2_ concentration in the product stream and will influence the choice between designing an electrolyte recycling system or a single-pass system. High flow velocities would require a recycling system in order to achieve a sufficiently high H_2_O_2_ concentration, but will allow for O_2_-resaturation and maintain higher O_2_ concentrations along with the increasing H_2_O_2_ concentration. Lower flow velocities will be necessary in a single-pass system, but such a system will have to cope with decreasing the O_2_ concentration during the same H_2_O_2_ concentration increase needed to satisfy application requirements. Therefore, the electrode height and flow velocity should be optimized to fit the current density and system requirements as well.^20,30^ Alternatively, the inlet O_2_ concentration can be increased by pressurizing the system, or options for in-channel saturation, such as bubbling O_2_ directly into the reaction channel, could be investigated.

Although the low O_2_ solubility remains a challenge in any aqueous system, the flow-through configuration allows for higher current densities and thicker channels with larger total amounts of available O_2_ compared to a flow-by configuration, while avoiding stability issues in GDEs due to salt formation,^31^ flooding,^32^ and water management in general.^33,34^ Even so, flow-through and GDE configurations are promising concepts to solve the limitation of the O_2_ mass transfer, each with their own advantages and drawbacks. Although the stability issues in GDEs need to be considered, GDEs provide better O_2_ availability in the entire channel compared to a flow-through system, whereas the flow-through system might offer easier scalability and higher stability due to the more simple and robust system design.

## Conclusions

We electrochemically produced H_2_O_2_ via the two-electron ORR with Cu-tmpa as a homogeneous catalyst at neutral pH in an electrochemical flow cell with different electrode configurations. We achieved similar Faradaic efficiencies for H_2_O_2_ to previously studied RDE systems with the Cu-tmpa catalyst, while the electrode area can be extended in flow cells. This indicates that this is a promising way of scaling up this reaction. We achieved the highest current density and H_2_O_2_ concentration in a flow-through configuration. The limiting current density was improved 10–25 times in the flow-through compared to the flow-by configuration due to the larger electrode area and due to higher mass transfer coefficients in flow-through electrodes. The electrode configuration had a significantly larger effect on the limiting current than the electrolyte flow rate; the flow velocity has only a minor effect when using a small (3.4 cm long) flow-through electrode. The high current density in flow-through electrodes was paired with increased difficulties in maintaining the initial current density and FE during CA. The higher O_2_ consumption- and H_2_O_2_ production rates shifted the [O_2_]/[H_2_O_2_] ratio in favor of H_2_O_2_ reduction and increased the competition between O_2_ and H_2_O_2_ reduction over time. Although this resulted in the most severe performance drop in the flow-through configuration, the H_2_O_2_ production rate remains the highest in this configuration, and the H_2_O_2_ concentration entered the mM range. In contrast, the suspension electrodes, which have an even higher contact area with the liquid phase, did not yield any detectable H_2_O_2_. We showed that any H_2_O_2_ that may be produced will be immediately decomposed by the AC material, rendering this particular carbon material unsuitable for the ORR to H_2_O_2_.

We have demonstrated that implementing a flow-through principle in an ORR flow system can greatly reduce mass transfer limitations already at low electrolyte flow and boost H_2_O_2_ production rates, enabling us to produce meaningful H_2_O_2_ concentrations in a neutral solution with Cu-tmpa as the ORR catalyst. Although the availability of O_2_ is greatly improved on the microscale, it will decrease through the height of the channel and cause difficulties when scaling to larger flow cells. Here, increasing the flow rate will have a larger positive effect than that in the small flow cell used in the experiments. Future studies should apply a fast O_2_ saturation method to ensure the maximum O_2_ concentration at the channel inlet and carefully design the electrode height, flow velocity, and recirculation ratio to suit the current density in the electrochemical H_2_O_2_ synthesis systems.

## Supporting Information Available

Schematic of ORR flow cell and setup; detailed description of electrochemical analysis; additional CV scans of suspension electrodes; calculation of overall O_2_ depletion over time; used method for estimating limiting currents and predicting them from Sherwood correlations; and estimation of pressure drop in the flow-through system (relevant data available in the Zenodo repository at 10.5281/zenodo.12622235) (PDF)
