# Supplementary material for: 20-Fold Increased Limiting Currents in Oxygen Reduction with Cu-tmpa by Replacing Flow-By with Flow-Through Electrodes
Source: ACS Sustain Chem Eng. 2024 Aug 12;12(34):12909–18. doi: 10.1021/acssuschemeng.4c03919 (PMC11351704; doi:10.1021/acssuschemeng.4c03919)
Supplement: Supplementary file 1 — sc4c03919_si_001.pdf [file sc4c03919_si_001.pdf]

Supporting Information

Twentyfold increased limiting currents in oxygen reduction with Cu-tmpa by replacing flow-by with flow-through electrodes

Nathalie E.G. Ligthart[1], Phebe H. van Langevelde[2], Johan T. Padding[3], Dennis G.H. Hetterscheid[2], David A. Vermaas*[1]

[1] Department of Chemical Engineering, Delft University of Technology, Delft, The Netherlands

[2] Leiden Institute of Chemistry, Leiden University, Leiden, the Netherlands

[3] Department of Process and Energy, Delft University of Technology, Delft, The Netherlands

Email: D.A.Vermaas@tudelft.nl

Contents

1 Experimental 1

1.1 ORR flow cell and setup 1

1.2 Electrochemical analysis 2

2 Catalyst concentration and stability..... 2

3 Calculation of overall O2 depletion..... 3

4 Comparing limiting currents to Sherwood correlations..... 3

4.1 Estimating limiting currents..... 3

4.2 Predicting limiting currents from Sherwood correlations..... 4

5 Pressure drop in the flow-through system 6

1 Experimental

1.1 ORR flow cell and setup

We used the custom-made PMMA electrochemical flow cell shown in Figure S1a to perform all ORR experiments. The flow cell consists of two backplates and two 3 mm thick flow channels, separated by a membrane. Both backplates have an inlay for the current collector or anode with leak-tight connections for two electrical connections from the back. The flow channels have an inlet designed to fit a micro reference electrode. We make use of two electrolyte reservoirs, which are connected to the electrochemical cell and peristaltic pumps as shown in Figure S1b. The catholyte reservoir is sparged with pure O_2 before and during the experiment, and is open to the air to allow for easy liquid sampling.

The glassy carbon foam is slightly thicker than the flow channel (3.2 versus 3.0 mm) to ensure good electrical contact with the current collector plate by pressing the membrane on top of the foam in between the channels. The glassy carbon plate was cleaned by polishing with 1 and 0.5 µm alumina slurries, rinsed and finally sonicated in deionized (DI) water for 16 minutes. A new piece of foam (24 by 34 mm) was cut before each experiment. A small hole (Ø3 mm) was punched in the foam at the RE inlet to accommodate RE insertion. The foam was cleaned by rinsing with DI water, sonicating in DI water for 16 minutes, and blow-drying with a nitrogen stream until no more water emerged.

The used foam had dimensions of 24x34x3.2 mm and a specific surface area of 3937 m^2/m^3 (specification provided by Goodfellow), resulting in surface area of 102 cm^2, and a total surface area (plate + foam) of 110 cm^2 in contact with the electrolyte. This is a factor of 13.5 times larger than the contact area of only the plate (8.16 cm^2) used in the flow-by configuration.

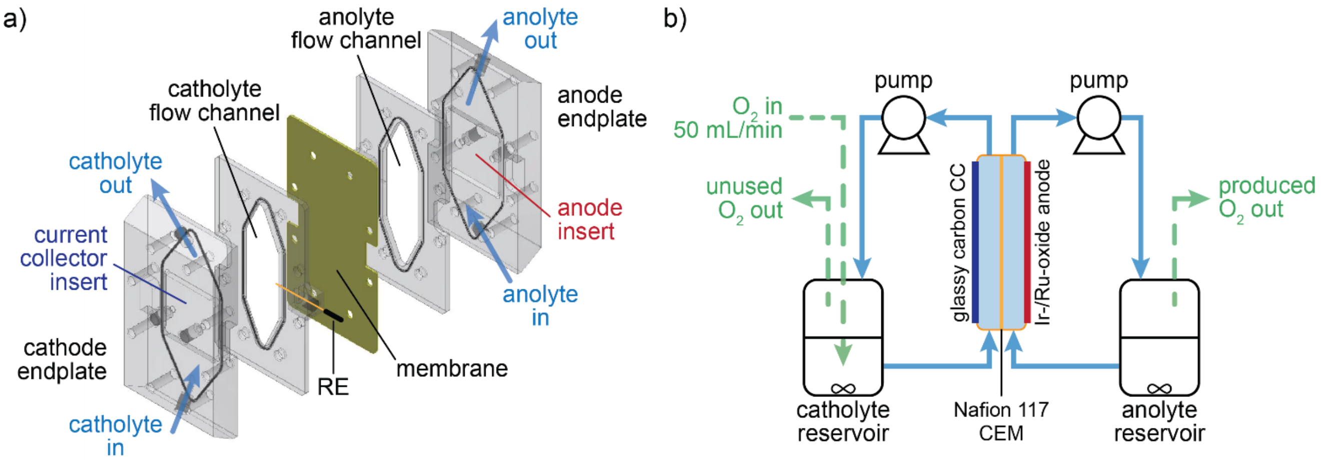

Figure S1. Illustration of the a) flow cell and b) setup used for the ORR experiments. Figure S1a is reproduced from the Supplemetary Information of Ref. (1) with permission from the Royal Society of Chemistry.

1.2 Electrochemical analysis

Cyclic voltammetry scans (CVs) were performed before each chronoamperometry (CA) scan to investigate the activity of the current collectors, the influence of Cu-tmpa concentration, and flow speed. In addition, CVs were measured after each CA to check the stability of the system. We scanned between -0.4 and 1.1 V vs. RHE, at a step size of 10 mV and scan rate of 100 mV/s, for at least 3 cycles. We used a slightly larger voltage window in some cases, mostly in the suspension electrodes, to make sure that the reaction was not occurring at a higher potential.

We performed 1 hour of CA to evaluate the oxygen to H_2O_2 reaction performance in each electrode configuration. We ran each CA at the working electrode potential resulting in roughly 75% of the peak current, as determined from the CVs. No catalytic current was visible in the CV measurements with the suspension electrodes because they were dominated by the capacitive current. We therefore ran the suspension CAs at similar potentials as we did for the flow-by and flow-through configurations. The precise conditions of each experiment are listed in Table S1.

Table S1. Reaction conditions of the ORR experiments.

Cell configuration	Applied E vs. RHE	Flow rate (mm/s)
**Flow-by**	0.31	19
**Flow-through**	0.21	19
**Suspension – 10 wt% AC**	0.31	9
**Suspension – 20 wt% AC**	0.21	9

2 Catalyst concentration and stability

CV scans of 10 (Figure S2a) and 20 wt% (Figure S2b) AC suspension electrodes with various concentrations of Cu-tmpa and O_2-saturated electrolyte have been recorded. Scans were again performed before and after CA to check the influence of Cu-tmpa concentration and the stability during catalysis. No influence of Cu-tmpa concentration is visible in either of these scans. The post catalysis scans are notably less steep than the scans taken before catalysis. This can indicate a loss of capacitive current due to partial charging of the Electric Double Layer Capacitance (EDLC) during the CA, or a loss in conductivity due to suspension sedimentation.

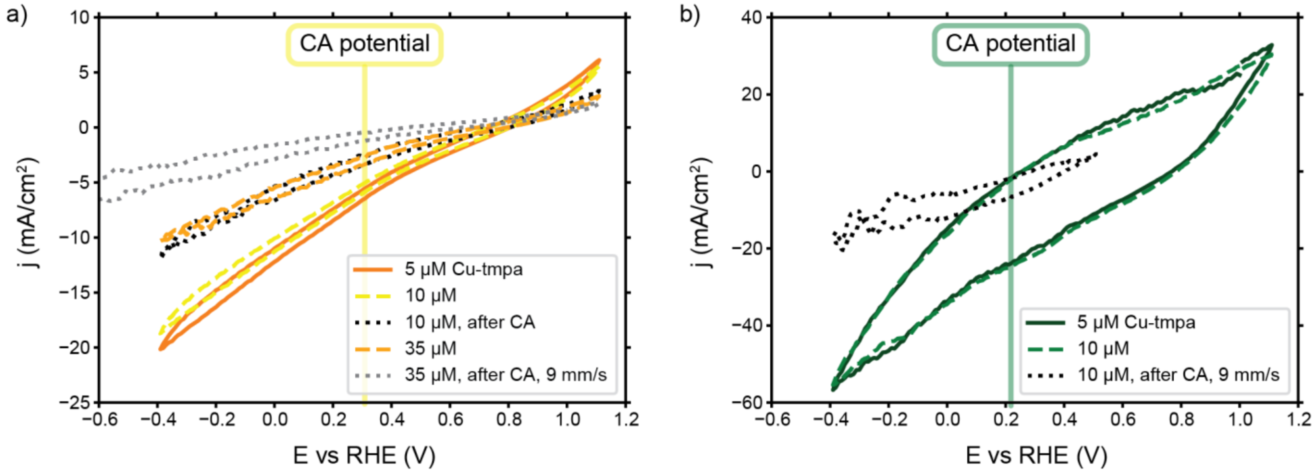

Figure S2. Influence of Cu-tmpa concentration and 1 hour of reaction conditions on CV scans of Cu-tmpa with a a) 10 wt% AC suspension, and b) 20 wt% AC suspension as current collectors. The potential at which chronoamperometry (CA) was performed is indicated in the panels, and all scans were performed under a flow rate of 19 mm/s, unless indicated otherwise.

3 Calculation of overall O_2 depletion

Taking a catholyte volume of 80 mL and assuming maximum O_2 saturation of 1.1 mM at the start of the experiment, we have 0.09 mmol of O_2 available when starting the reaction. Taking the average currents during the flow-by (-0.3 mA/cm^2, -2 mA) and flow-through (-3.8 mA/cm^2, -31 mA) operations, and assuming that all current consumes O_2, we can estimate the O_2 consumption. The O_2 concentration through time, when no fresh O_2 is supplied, is shown in Figure S3 for both configurations. It is clear that the flow-through configuration will consume all O_2 within about 9 mins and needs a fast resaturation technology to keep up with the O_2 consumption, while this is significantly less critical in the flow-by configuration. The latter can run for 60 mins without consuming even half of the O_2 starting concentration.

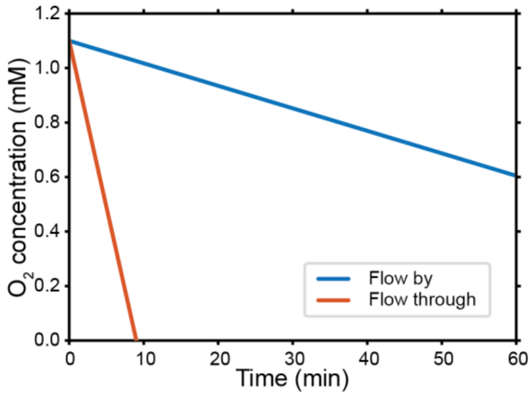

Figure S3. Estimated decreasing O_2 concentration in the bulk electrolyte in case of the flow-by and flow-through configurations.

4 Comparing limiting currents to Sherwood correlations

4.1 Estimating limiting currents

We estimated the limiting current in each CV scan by performing two linear fits and calculating the current at the intercept. The first line was fitted in the mass-transfer limited region (between -0.39 and -0.09 V vs RHE), the second line was fitted in the steep region close to the reaction potential of 0.3 V vs RHE (between 0.26-0.36 V vs RHE in case of the flow-through, and between 0.11 and 0.31 V vs RHE in case of the flow-by case). An example is shown for both a peak-shaped CV and for an S-like CV in Figures S4. The current density is then calculated from the measured current and the geometrical area of the current collector.

The error bars in Figure 3 are an indication of the maximum error caused by our choice of potential window. We estimated the errors at 10 and 14% of the calculated current density for the flow-by and the flow-through cases, respectively. We obtained these values for the errors by performing the fits over large and small potential windows and calculating the standard deviation of in current density from the intersects of all combinations of fits for one CV scan per experiment.

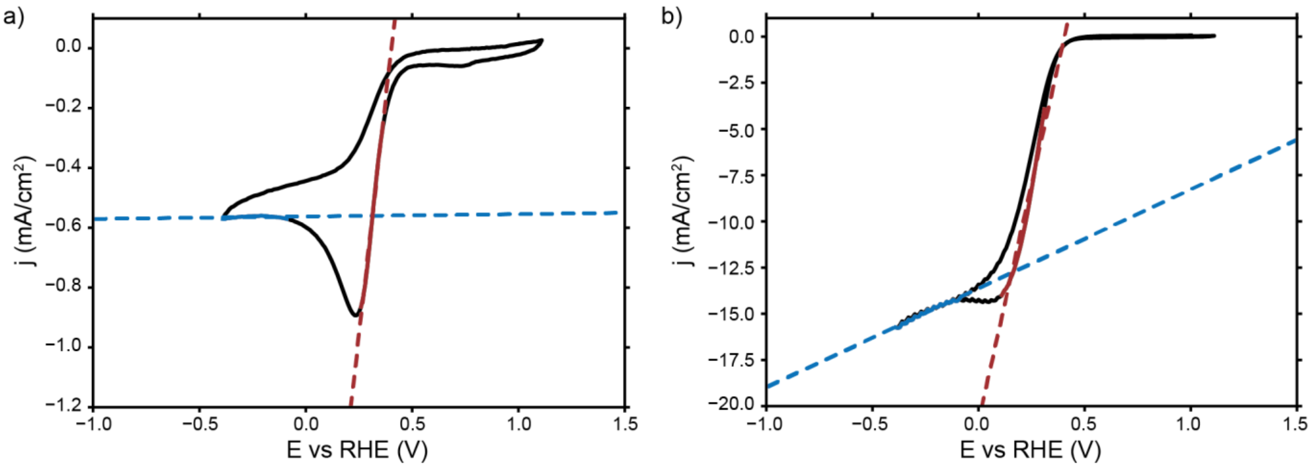

Figure S4. Typical CV scans in the a) flow-by and b) flow-through configurations. We estimated the limiting current densities by calculating the intercept between linear fits in the mass transfer-limited region and the region around 0.3 V vs RHE.

4.2 Predicting limiting currents from Sherwood correlations

Here, we give the complete derivation of the local O_2 concentration inside the flow-through (foam) electrode, all input parameters are given in Table S2. We start with the microscopic mass balance

$$\frac{d}{dt}(c(x)BWdx) = BWuc|_x - BWuc|_{x+dx} - kA_v c(x)BWdx \tag{S1}$$

and assume steady state to arrive at

$$0 = uc(x) - uc(x+dx) - kA_V c(x)dx \tag{S2}$$

$$u\big(c(x+dx) - c(x)\big) = -kA_V c(x)dx \tag{S3}$$

$$udc = -kA_V c(x)dx \tag{S4}$$

$$u\frac{dc}{dx} = -kA_V c(x) \tag{S5}$$

$$\frac{dc}{dx} = -\frac{kA_V}{u}c(x) \tag{S6}$$

We impose the boundary condition $c(0) = c_0$ $(= c_{bulk})$ to get the concentration profile, which is dependent on the inlet concentration (c_0), the mass transfer coefficient (k), the specific area of the foam (A_V), the flow rate (u), and the location (x).

$$c(x) = c_0 e^{-\frac{kA_V}{u}x} \tag{S7}$$

The local geometric current density is then given by

$$j_{loc}(x)A = nFkA_V c(x)V \tag{S8}$$

$$j_{loc}(x)Bdx = nFkA_V c(x)BWdx \tag{S9}$$

$$j_{loc}(x) = nFkA_V c(x)W = nFkA_V W c_0 e^{-\frac{kA_V}{u}x} \tag{S10}$$

Which can be integrated to find the average geometric current density

$$j_{avg}^{foam} = \frac{1}{L}\int_0^L j_{loc}(x)dx = \frac{nFkA_V W c_0}{L}\int_0^L e^{-\frac{kA_V}{u}x}dx = \frac{nFWc_0 u}{L}\left(1 - e^{-\frac{kA_V L}{u}}\right) \tag{S11}$$

We used equations S7 and S11 to create Figure 7 in the manuscript.

The situation in the flow-by configuration is simpler because the O_2 consumption is significantly lower and we can assume that the bulk concentration is constant along the complete height of the channel $(c(x) = c_0)$. This gives us an average geometric current density over the plate electrode of

$$j_{avg}^{plate} = nF\mathrm{k}^{plate}c_0 \tag{S12}$$

This is equal to the limiting current on the flow-by electrode (j_{lim}^{plate}).

The expected limiting current on the flow-through electrode can be calculated in a similar manner, i.e. by assuming that the bulk stays O_2-saturated ($c_0 = 1.1$ mM) everywhere in the foam, and results in

$$j_{lim}^{foam} = nFk^{foam}c_0 \qquad (S13)$$

Comparing the limiting current on the flow-by and flow-through electrodes gives an improvement factor of $j_{lim}^{foam}/j_{lim}^{plate}$.The predicted improvement factor and the experimental results are shown together in Figure S5. They are in good agreement at a factor of 19.5-16 for the theoretical values, and 25-15 for the experimental values. The decrease in improvement with increasing flow rate follows from the weaker flow rate-dependence of the Sherwood number in the flow-through versus the flow-by configuration.

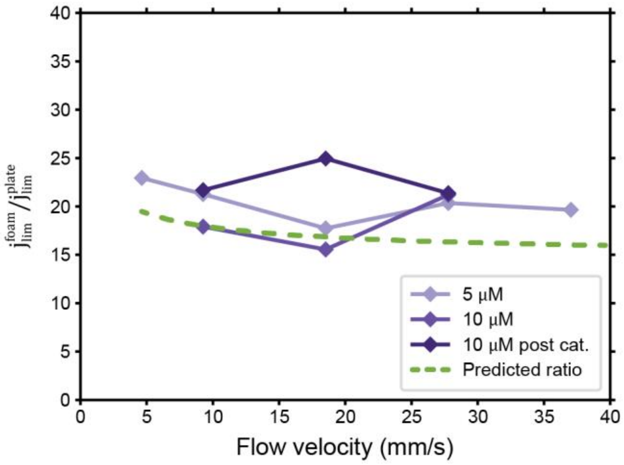

Figure S5. The factor ($j_{lim}^{foam}/j_{lim}^{plate}$) by which the limiting current density is increased in the flow-through compared to the flow-by configuration per flow rate during our experiments (solid purple lines) and what we predict from Sherwood correlations (green dashed line).

Table S2. Input parameters for Sherwood correlations and mass transfer coefficient extraction.

**Shared – independent of configuration**		
$\boldsymbol{L}$	$34 \cdot 10^{-3}$ m	electrode length
$\boldsymbol{B}$	$24 \cdot 10^{-3}$ m	electrode width
$\boldsymbol{W}$	$3 \cdot 10^{-3}$ m	electrode-membrane spacing, channel depth
$\boldsymbol{u}$	Experiment: $18.6 \cdot 10^{-3}$ m/s	(superficial) flow velocity
$\boldsymbol{\nu}$	10^{-6} m²/s	kinematic viscosity
$\boldsymbol{D}$	$2 \cdot 10^{-9}$ m²/s	diffusion coefficient O_2 in water
$\boldsymbol{n}$	2	electrons transferred in reaction
$\boldsymbol{F}$	96485 C/mol	Faraday constant
$\boldsymbol{c_{bulk}}$	1.1 mol/m³	maximum O_2 concentration

Configuration specific			
Flow-by		**Flow-through**	
$\boldsymbol{\gamma = \frac{B}{L}}$	0.71	ε	0.965, porosity
$\boldsymbol{d_c = d_h = 2BW(B+W)}$	$5.33 \cdot 10^{-3}$ m, hydrodynamic diameter	$d_c = d_s$	$200 \cdot 10^{-6}$ m, typical strut size
$\boldsymbol{A}$	$8.16 \cdot 10^{-4}$ m², electrode area	A_V	4219 m²/m³, specific electrode area

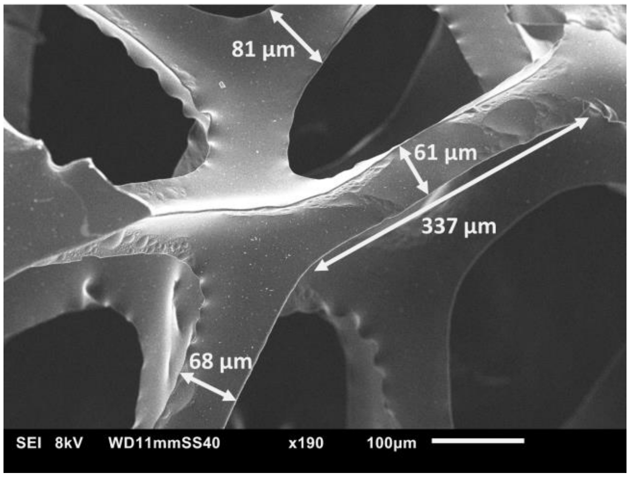

Figure S6. SEM image of the 60 ppi glassy carbon foam and length measurements used to estimate a typical strut size.

5 Pressure drop in the flow-through system

The pressure drop in an ideal case of an infinitely wide channel with fully developed laminar flow can be estimated from the Darcy-Weisbach equation(2) (eq. S14)

$$\Delta p = \frac{12\mu L v}{h^2} = \frac{12\mu L^2}{t_{res}\frac{1}{4}d_h^2} \tag{S14}$$

in which μ is the viscosity of water (Pa·s), L is the cell length (m), v is the flow velocity (m/s), h is the channel thickness (m), t_{res} is the residence time (s), and the hydraulic diameter (d_h) can be calculated from(3, 4)

$$d_h = \frac{4\varepsilon}{\frac{2}{h} + \frac{(1-\varepsilon)S_{CC}}{V_{CC}}} \tag{S15}$$

to account for the presence of spacers or, as in our case, the presence of a foam. Here, ε is the foam porosity, S_{CC} is the surface (m^2) of the porous current collector, and V_{CC} is the solid volume (m^3) of the foam. Figure S7 shows the theoretical pressure drop over a 50 cm long foam at various flow rates.

Assuming a current density of 20 mA/cm^2 and a cell voltage of 1.5 V, an electrolyzer of 50 cm long, with a 0.3 cm thick channel, and 1 cm wide electrode, would use 1.5 W of power to perform the electrochemical reaction, the required pumping power (P_{pump}) can be calculated with

$$P_{pump} = \frac{\Delta p Q}{\eta} \tag{S16}$$

where a flow velocity of 600 mm/s gives a theoretical pressure drop of 0.2 bar and a flow rate (Q) of 18 mL/s. Assuming a pump efficiency (η) of 70% gives a pumping power of 0.51 W, which is already 34% of the utilized electrochemical power.

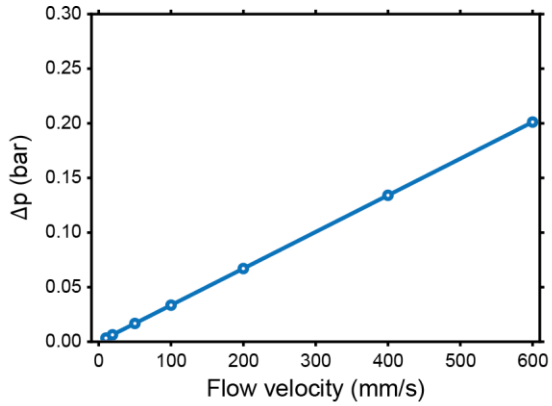

Figure S7. Calculated pressure drop in a flow-through cell for the ideal case of an infinitely wide channel with fully developed laminar flow.

REFERENCES

1. Ligthart NEG, Prats Vergel G, Padding JT, Vermaas DA. Practical potential of suspension electrodes for enhanced limiting currents in electrochemical CO2 reduction. Energy Advances. 2024.
2. Vermaas DA, Guler E, Saakes M, Nijmeijer K. Theoretical power density from salinity gradients using reverse electrodialysis. Energy Procedia. 2012;20:170-84.
3. Schock G, Miquel A. Mass transfer and pressure loss in spiral wound modules. Desalination. 1987;64:339-52.
4. Da Costa AR, Fane AG, Wiley DE. Spacer characterization and pressure drop modelling in spacer-filled channels for ultrafiltration. Journal of Membrane Science. 1994;87(1):79-98.
